# How fear of intimacy affects infertile men’s neuropsychological functioning through mental toughness

**DOI:** 10.3389/fpsyt.2023.1049008

**Published:** 2023-07-24

**Authors:** Sajid Hassan, Mazhar Iqbal Bhatti, Shazia Habib, Sidra Fatima, Sher Bhader, Nazeer Hussain Khan, Enshe Jiang

**Affiliations:** ^1^Institute of Nursing and Health, Henan University, Kaifeng, China; ^2^Department of Psychology, International Islamic University, Islamabad, Pakistan; ^3^Department of Applied Psychology, Government College University Faisalabad, Faisalabad, Pakistan; ^4^University Gillani Law College, Bahauddin Zakariya University, Multan, Pakistan; ^5^Department of Animal Sciences, Quaid I Azam University, Islamabad, Pakistan

**Keywords:** fear of intimacy, quality of life, neuropsychological impairment, mental toughness, infertility

## Abstract

**Objective:**

There is a significant need in Pakistan to investigate the psychological effects of infertility on the mental health of infertile men. The current study examined how fear of intimacy affects neuropsychological impairment and evaluated its relationship to other variables including quality of life and mental toughness.

**Method:**

An analytical cross-sectional study was carried out on infertile male patients in various healthcare settings in Punjab, Pakistan. The participants were recruited using a non-probability (purposive) sampling strategy. The sample size was 120 infertile. SPSS 26 was used to analyze the data.

**Results:**

Fear of intimacy was found significant impact on neuropsychological impairment (*r* = 0.40; ***p < 0.001), as well as fear of intimacy, significantly associated with emotional problems (*r* = 0.48; **p < 0.01), learning problems (*r* = 0.33; **p < 0.01), sensory and motor problem (*r* = 0.55; **p < 0.01), concentration problem (*r* = 0.21; **p < 0.01), mental & physical in coordination (*r* = 0.37; **p < 0.01) and depression (*r* = 0.22; **p < 0.01). Fear of intimacy has negative impact on QoL (*r* = −0.25; *p > 0.05). Similarly, neuropsychological impairment was found to be negatively associated with QoL (*r* = −0.52; **p > 0.01). The relationship between fear of intimacy and neuropsychological impairment was found to be significantly mediated by QoL. Furthermore, the findings revealed that mental toughness significantly moderated the relationship between fear of intimacy and neuropsychological impairment.

**Conclusion:**

Overall, infertile men in Pakistan had relatively high levels of fear of intimacy, which largely caused neuropsychological impairment. This study can help neuropsychological researchers, mental health professionals, as well as policymakers in improving clinical mental health practices for infertile patients.

## Background of the study

Infertility is defined as the inability to conceive after 1 year of continuous, unprotected sexual activity and affects approximately one out of every five couples globally (1, 2). Around the world, many people between the ages of 15 and 49 are affected by infertility (3). According to a prior study, 15.5% of reproductive-age women in the USA experience infertility (4), 24.5% of women in France (5), and 25.5% of women in China (6). It is recognized that infertility is a global public health issue that affects both genders’ reproductive health (7). There may be between 60 and 80 million infertile couples (8, 9). Infertility is a sad journey for both genders (10, 11).

Male attributes constitute almost 50% of all cases, and nearly 7% of men worldwide are affected by this status (12). Moreover, it has been extensively established that infertility has a substantial influence on both spouses’ mental well-being, with difficulties such as poor self-esteem, sexual misery, miserable, guilt, panic, frustration, and interpersonal troubles between them. Moreover, some studies have found that these negative feelings can be harmful to personal health, marital satisfaction, relationship quality, and even pregnancy, leading to a vicious cycle (13). Potentially negative dose–response associations between stress (14) and unhappiness (15) and sperm quality have been discovered in males (concentration, motility, and total sperm count). Man’s reproductive dysfunction, a significant leading contributor to male infertility, is intimately linked to mental illness (16, 17). Management of infertility is intimately tied to a patient’s mental condition, and coping with infertility has a considerable negative influence on their psychological well-being and general happiness. These might result from two underlying factors: the significance of motherhood in a couple’s lives and the connection between fertility and patients’ cognitive dysfunction as psychological (social, relational, and marital) experiences.

## Theory and development of hypotheses

Coping theory was used in this investigation to assess the hypothesized effects. Coping is described as “the neurocognitive and behavioral efforts expended to handle certain external and/or internal pressures which are evaluated as surpassing the human’s capabilities (18).” Coping refers to the adaptive activities taken by an individual in reaction to a damaging illness that impacts his or her lifestyle (19). Inside this contextual model, the adapting hypothesis is the most extensively utilized and accepted in neuropsychology.

Ultimately, how patients cope is determined by the resources accessible to people (quality of life, neurocognitive, psychological, and behavioral) (20). Similarly, in the present study, during infertility illness, infertile patients would question themselves, “What is at risk for me in this scenario?” The main problem is determining the probable consequences of this occurrence (particular internal/external demands) and the personal awareness of the infertility disorder (18). There are two main categories of obstructive illnesses in monitoring: challenges, which are reported cases that are perceived to have positive outcomes (infertile patients will experience low fear of intimacy levels in this study due to infertility), and affects, which are incidents that are interpreted to have negative adverse consequences) (due to high fear of intimacy patient will face the problems of neuropsychological impairment such as). People frequently evaluate their coping choices (mental toughness as an internal resource) in addition to judging the seriousness of disease. Infertile patients make decisions on their amount of control over the circumstance and what they feel should be done about it based on the coping strategies that are accessible to them. As previously stated, coping theory is suited for analyzing the predicted model since it tackles the complete process of how these infertile individuals would adapt to an illness associated with infertility.

## Fear of intimacy, QoL, and neuropsychological impairment

In addition to anxiety, sadness, social isolation, and diminished libido, it can cause marital conflict, a sense of helplessness, guilt, humiliation, and worthlessness (21, 22). Studies by Onofre et al. (23) and Bhamani et al. (22) claim that “male factor” infertility accounts for 35% to 50% of cases of infertility in South Asian low and middle-income nations (LMIN). Tragically, societal and cultural factors affect how society views infertile patients. In our patriarchal society, infertile partners who are unable to conceive a child are frequently the target of discrimination and shame (24). As a result, infertile women are left out of social and traditional rituals (25), are physically, emotionally, and verbally mistreated, and frequently get divorced even if the primary problem is with men (25, 26). Thus, infertile individuals who are accused of being childless experience emotional sadness, frustration, poor mental health, and social dysfunction, which may harm their marital relationship (fear of intimacy), as well as their quality of life (27, 28). The phrase “fear of intimacy,” sometimes known as “intimacy avoidance,” refers to a fear of having intimate emotional or physical contact with another person (29). According to some scholars, both intimacy avoidance and marital issues are associated with psychological illnesses (30).

Infertility can have an impact on an individual’s neuropsychological functioning (31), which may lead to neuropsychological impairment. According to the Diagnostic and Statistical Manual of Mental Illnesses, Fifth Edition (DSM-5 American Mental Association 2013), neuropsychological impairment symptoms in psychiatric disorders are characterized by impaired decision-making, attention, memory, and executive function. One of the most prevalent and enduring signs of depressive disorders is neuropsychological impairment. According to Cha et al. (32), neuropsychological impairment is found in more than 90% of patients with mental disorders (MDD). Based on the foregoing data, we can assume that male infertiles’ fear of intimacy (losing marital relationships) may cause their neuropsychological impairment symptoms to worsen over time.

Numerous physiological, psychological, and social effects, such as grief, anxiety, stigma, and social isolation, can have a detrimental influence on the QoL of those who are suffering from infertility (33, 34). QoL is defined similarly by the WHO as “individuals’ judgments of their place in life in respect to their aims, aspirations, standards, and worries, as well as in the context of the culture and value systems in which they live” (35). Fertility QoL thus provides a comprehensive picture of the life circumstances of infertile patients during their infertility period (36, 37). Numerous studies have revealed that the quality of life (QoL) of infertile women was lower than that of their fertile counterparts. Furthermore, it has been shown that low reproductive QoL in infertile women is negatively correlated with medication compliance (38) and may lead to mental dysfunction (39). Various researchers claimed women’s infertility has been connected to a lower quality of life (35, 40). Furthermore, based on Zurlo et al.’s (41) research, we can assume that QoL is an important factor that influences infertile men’s intimacy-related fear as well as their neuropsychological impairment.

In the same way, infernality may affect our cognitive abilities such as control and confidence (42). Similarly, these cognitive abilities close link with mental toughness. Furthermore, mental toughness is a psychological trait that governs how people react to pressure, difficulty, and discomfort independent of their environment (43). The development of psychological resilience can be aided by mental toughness, which can shield against the detrimental psychological impacts of infertility (31). Strong self-esteem, optimism, self-confidence, problem-solving skills, and a happy existence are all traits that are correlated with mental toughness. Thus, it appears that mental toughness is the key to enhancing patients’ quality of life during stressful situations (44, 45). Mentally tough people are more likely to perceive their environment as within their control, to believe they are capable and influential, to stick with their goals even when things go challenging and to see problems as opportunities for growth (46). In line with all of the preceding points, we propose our research model reveals how fear of intimacy effect infertile men’s neuropsychological impairment through mediating influence of QoL as well as how mental tough buffers the effect of fear of intimacy on neuropsychological impairment. In light of the preceding literature, the hypotheses of this investigation were as follows:

*H1.* Fear of intimacy is positively related to neuropsychological impairment.

*H2.* Fear of intimacy is negatively related to quality of life.

*H3.* Neuropsychological impairment is negatively related to Quality of life.

*H4.* The indirect association between fear of intimacy and neuropsychological impairment is mediated by quality of life.

## Moderating role of mental toughness

Mental toughness reduces the negative psychological effects of disease (47). Mental toughness and mental health are positively associated (48). According to current studies, establishing mental toughness is one approach to cope with the psychological health challenges brought on by infertility disease (47). For example, Jones and Parker (49) discovered that higher levels of mental toughness were connected with lower risk factors in terms of the repercussions of the illness that causes infertility. A different study found that persons with higher mental toughness are less likely to become ill (50). Similarly, individuals with poorer mental toughness had more difficulty dealing with the situation’s emotional obstacles. According to the primary component of the coping theory, this study interprets mental toughness, which is a psychological resource for infertile patients that helps them cope with cognitive impairment. According to this study, persons with low mental toughness are more vulnerable to stresses associated with their disease that risk their mental health. These people, who think they lack the ability to satisfy their cognitive demands, are likely to be the most alarmed by the possibility of neuropsychological impairment and QoL. Those with high levels of mental toughness, on the other hand, would suffer less overall neuropsychological impairment and would be less affected by new pressures. This demonstrates that mental fortitude can mitigate the particular relationship between fear of intimacy and neurocognitive impairment. As a result, we proposed the following hypotheses:

*H5.* The indirect association between fear of intimacy and neuropsychological impairment is moderated by mental toughness.

## Methods

### Study design and data collection

From December 2021 to May 2022, we conducted a cross-sectional study in Punjab Province, Pakistan. To test hypotheses, the participants were recruited from various healthcare departments. Infertile patients over the age of 18 who had been diagnosed with infertility and could communicate fluently in Urdu were eligible to participate. Infertile patients who were currently infertile with a history of other psychiatric illnesses and cognitive impairments, as well as infertile women, were excluded from the current study. The study encouraged all eligible participants to take part. The researchers confirmed that the participants were well-informed about the study’s goal and procedures. After receiving written informed permission, individuals were asked to complete a structured questionnaire. A closed-ended structured questionnaire with a maximum Likert scale of 1 to 5 (where 1 = strongly disagreed and 5 = strongly agreed) was utilized in the current study, which was first created in English. Respondents were informed that participation was completely voluntary and that they could withdraw from the study at any point during the data collection process. Ethical approval was obtained from the relevant departments, and the responses of the participants were kept secret from any participating firm executive. The survey had 174 participants in total, but the authors only received 133 sets of completed questionnaires, 13 questionnaire sets were eliminated from this study because they were insufficient, leaving 120 questionnaire sets that could be used and had a response rate of 68.96%.

## Measures

### Fear of intimacy with helping professional scale (FIS-HP)

The FIS-HP scale has 18 items. The FIS-HP is being developed to measure an individual’s level of comfort in discussing personal feelings with a professional partner. The items are evaluated on a 5-point scale ranging from 1 (not at all indicative of me) to 5 (extremely characteristic of me), with a total score ranging from 18 to 90. Individuals with high FIS–HP scores indicate fear of sharing, anxiety over close sharing, and fear regarding intimacy. It has a high level of convergent validity. Cronbach’s alpha is 0.88, showing sufficient internal consistency (51).

### Mental toughness questionnaire (MTQ 10)

The MTQ-10 is a short summary of the MTQ-18 that includes the most valuable items in each of the four 4Cs dimensions (i.e., challenge, commitment, control, and confidence). “I generally handle any problems that arise well,” for example. The MTQ-10, like the previous MTQ, assesses a five-point Likert scale, with 1 representing “disagree” and 5 representing “agree.” It assesses the 4Cs as well as provides an overall score of mental toughness (control, commitment, challenge, and confidence). Even though the MTQ-10 has shown promising predictive validity. Cronbach’s alpha is 0.76, and the MTQ-10 has a satisfactory reliability score of 0.77 (52).

### World Health Organization’s quality of life questionnaire (WHOQoL-BREF)

The 26-item WHOQOl-BREF self-assessment questionnaire addresses four aspects of QOL (psychological, physical, social, and environmental domains). A high Likert scale score on the WHOQOL five-point BREF indicates a high quality of life while a low score on this scale reveals low QoL. WHOQOL-BREF has a Cronbach value of 0.78, with higher scores indicating better quality of life (53, 54).

### Neuropsychological impairment scale (NIS)

The Neuropsychological Impairment Scale (NIS), which has 46 items, will be used in this study. The NIS is a four-point Likert Type Scale that ranges from 1 to 4 such as 1 = “never,” 2 = “sometimes,” 3 = “most of the time” and 4 = “all of the time.” The sum of the scores represents the infertile patient’s overall neuropsychological impairment. The potential range of NIS scores is 46–184. The Neuropsychological Impairment Scale (NIS) assesses emotional, learning, concentration, mental, and physical coordination issues (55). It has satisfactory validity and a 0.83 Cronbach value. NIS has six such subscales as Emotional problems, learning problems, sensory and motor problem, concentration problem, mental & physical in coordination and depression. These all subscales measure an individual’s Neuropsychological Impairment.

### Sociodemographic factors

A structured survey form was designed to collect initial information from applicants about their demographic variables such as age range, education level, socioeconomic status, family life, mode of treatment, treatment duration, and living style (see Table 1).

**Table 1:** Demographics variables of the study.

Variables	*N*	Percentage	Variables	*N*	Percentage
**Age**			**Duration of infertility**		
Young adult	53	44.2	1–5 years	23	19.2
Middle adult	39	32.5	6–10 years	21	17.5
Older adult	28	23.3	11–15 years	35	29.2
**Education**			16–20 years	38	31.7
Under Matric	29	24.2	Up to 20 years	3	2.5
Matric	25	20.8	**Profession**		
Intermediate	15	12.5	Private job	47	39.2
Graduation	36	30.0	Govt. job	52	43.3
Master	15	12.5	Business	21	17.5
**Family**			**SES**		
Nuclear	74	61.7	Low	44	36.7
Joints	46	38.3	Medium	38	31.7
**Mode of treatment**			High	38	31.7
Government	57	47.5			
Private	63	52.5			

*N* = 120.

### Ethical approval

The Institutional Ethical Review Committee of the International Islamic University approved the ethical permission (ERC No. IIUI /PSY MSCP 397-21) for conducting research and consent for data collection. Before the start of this study, the study participants signed a written informed consent form. We provided the study participants with complete confidentiality. Only the researchers had access to the data.

### Analytical strategy

In this study, a two-step approach (56) was utilized to test the hypothesized model, as well as SPSS-26 and AMOS-24 were applied for data analysis. The descriptive statistical analysis for the constructs is shown in Table 1. The common method bias (CMB) was also explored. This was done since obtaining data from a single source at the same time might produce problems for CMB in the research (57). To prevent CMB, this study used the Harman single factor assessment technique. If the first element accounted for more than half of the variance in the CMB, the study would have a serious problem. The unrotated analysis found that the first component explained 26.38% of the variance, showing that the CMB was not a significant concern in this research.

### Measurement model

The data was analyzed using SPSS 26 and AMOS-24 versions, as well as a structural modeling approach. In the behavioral sciences, structural modeling, a highly general statistical modeling tool, is frequently used (58, 59). The model’s fit was analyzed, and the categories of goodness-of-fit measurements, incremental fit measures, absolute fit measures, and parsimonious fit measures suggested by Khan et al. (60) were measured. *x*^2^ = 187.34, df = 141, RMSEA = 0.032, CFI = 0.968, SRMR = 0.039, NFI = 0.926, IFI = 0.943, and GFI = 0.919; all values were within the specified range; the findings indicate that the fit between the measurement model and the data set are satisfactory. To validate the survey questionnaire’s objective and scope, a series of tests, including maximum shared variance (MSV) and average shared variance (ASV), were used. Every loaded item exceeded the acceptable standard value of 0.60 (61). Cronbach’s alpha values range from 0.700 to 0.900 in Table 2, which is higher than the previous researchers’ suggested threshold value of 0.70 (61–63). Additionally, the composite reliability values, which ranged between 0.731 and 0.917, exceeded the predicted threshold of 0.70 established by Nunnally (63). The extracted average variance (AVE) ranged from 0.518 to 0.816, which was somewhat higher than the actual MSV (64, 65) and may be higher than the benchmark value of 0.50 recommended by Bagozzi (64). Furthermore, all ASV values appeared to be lower than MSV. By adding up the results, the convergent validity of the measurement model as an accuracy measuring technique was confirmed. Table 2 provided additional evidence of discriminant validity. As long as all of the AVE square roots were greater than their correlation, the model’s discriminant validity could be said to have been established.

**Table 2:** Psychometric properties of measurements.

Constructs	FL	α	CR	AVE	MSV	ASV	Skewness	Kurtosis
FI	0.842–0.939	0.900	0.917	0.789	0.048	0.028	−0.991	1.932
MM	0.669–0.781	0.748	0.740	0.541	0.044	0.007	−0.472	0.872
QoL	0.643–0.769	0.720	0.731	0.518	0.452	0.201	−0.673	0.243
NPI	0.752–0.833	0.811	0.826	0.693	0.491	0.242	−0.763	1.267

FL, factor loadings; CR, composite reliability; AVE, average variance extracted; ASV, average shared variance; MSV, maximum shared variance; QoL, quality of life; NPI, neuropsychological impairment. At the *p* < 0.001 level, all factor loadings are significant.

## Results

**Figure 1:**
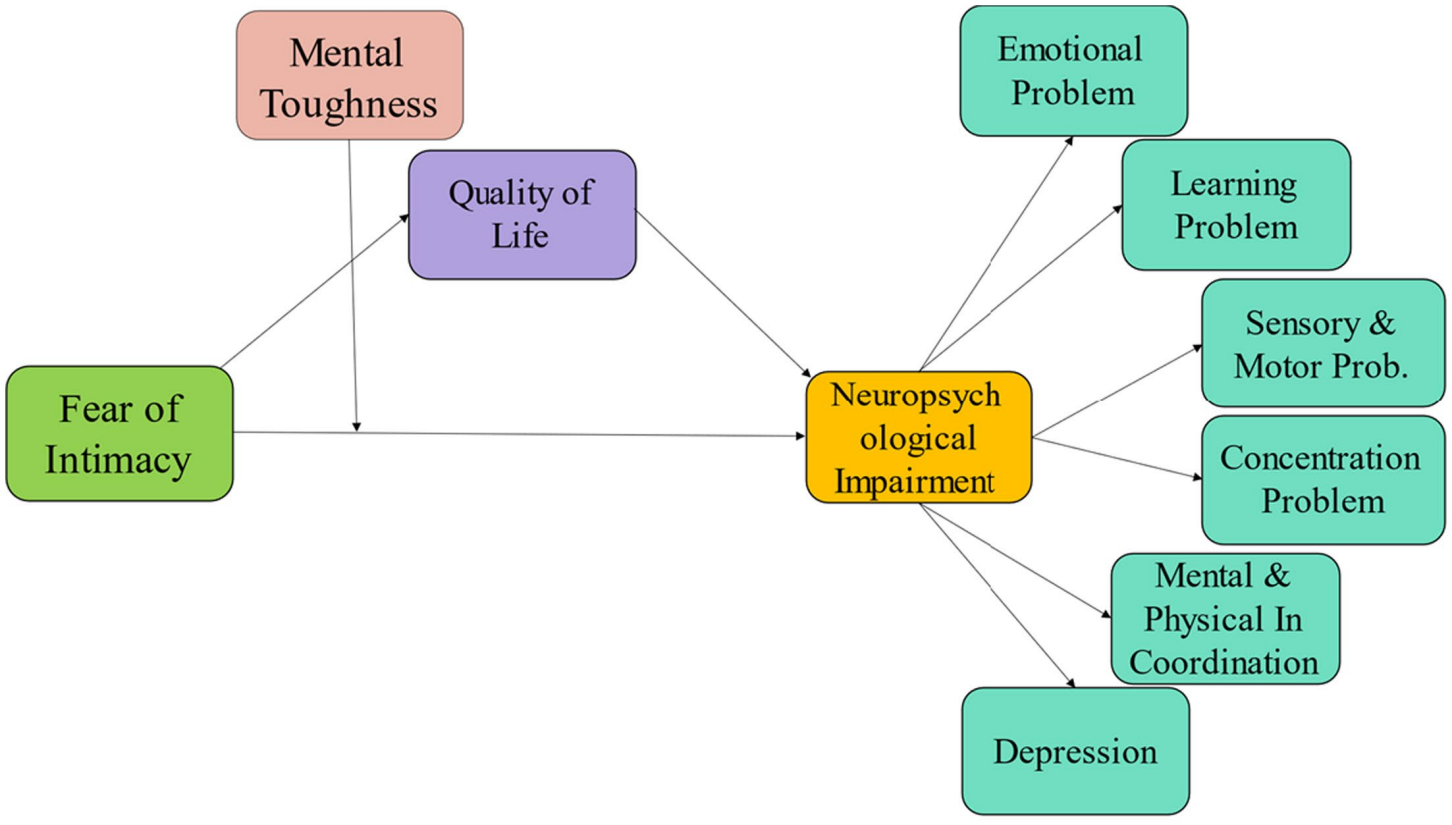
Research model.

Results from the structural model clearly show that the entire model is technically accurate because the values of *x*^2^/df = 1.95, RMSEA = 0.06, CFI = 0.96, GFI = 0.91, IFI = 0.95, and NFI = 0.92 are over the threshold value suggested by Tripp and Hair (66). Results reveal that, as depicted according to Figure 1, the majority of the hypotheses are supported. The results display that fear of intimacy has significant association with neuropsychological impairment (*r* = 0.40; **p < 0.01). H2: here fear of intimacy is significantly negative associated to QoL (*r* = − 0.18; *p > 0.05) and H3: that neuropsychological impairment has significant negative effect on the QoL (*r* = − 0.52; **p < 0.01) (Table 3).

**Table 3:** Variance estimates, means, standard deviation and intercorrelations matrix.

Variables	1	2	3	4	5	6	7	8	9	10	11
Fear of intimacy	–										
Mental toughness	−0.18*	–									
QoL	−0.25**	0.24**	–								
NPI	0.40***	−0.23**	−0.52**	–							
Emotional problem	0.48**	−0.39	−0.64*	0.34***	–						
Learning problem	0.33**	−0.52	−0.48**	0.41**	0.60**	–					
Sensory and motor	0.55**	−0.22	−0.17	0.27***	0.32**	28**	–				
Concentration problem	0.21**	−0.48*	−0.12*	0.61*	0.32*	0.38*	0.22**	–			
Mental and physical in coordination problem	0.37**	−0.63	−0.29*	0.55**	0.37**	58	0.30**	0.23*	–		
Depression	0.22***	−0.31	−0.12***	0.62***	0.40**	0.45**	0.20**	36**	0.23*	–	
*M*	64.85	18.85	23.80	96.76	32.39	48.33	13.04	19.77	14.32	10.21	–
SD	8.89	4.05	6.25	13.63	6.47	7.05	3.00	4.67	3.42	2.31	

*p < 0.05, **p < 0.01, ***p < 0.001. QoL, quality of life; NPI, neuropsychological impairment; M, means; SD, standard deviation. *n* = 120. Reliability information is on the diagonal. Variables are connected via group-mean centered correlations. A rough association was produced by averaging the factors. Correlation coefficients are shown in cells with a dash.

To examine how QoL mediated the relationship between fear of intimacy and neuropsychological impairment, we used the bootstrap approach. We used Milman’s et al. (67) bootstrap approach (bootstrap sample size = 5,000) to generate asymmetric confidence intervals (CIs) for the indirect association because the bootstrapped CIs approach generates asymmetric CIs for the indirect relationship using the respective distributions of the two regression coefficients that comprise the product term, it produces a more accurate estimate of the indirect relationship than conventional methods such as the Sobel test (67). Table 4 displays the findings of the mediating effects that QoL significantly mediated the relationship between fear of intimacy and neuropsychological impairment because CL (−1.31, −0.65) did not contain zero, continuing to support H4.

**Table 4:** Bootstrap results of the direct effects of fear of intimacy on outcome variables and moderating role of mental toughness.

Direct and indirect effects of fear of intimacy on NPI			*Β*	LLCI	ULCI
Fear of intimacy → Neuropsychology impairment			0.62***	0.21	0.67
Fear of intimacy → QoL → NPI			−0.45***	−1.31	−0.65
Regression analysis					
Variables	Model 1	Model 2	Model 3	Model 4	
Age	−0.36**	−0.29**	−0.20**	−0.14**	
Education	0.11	−0.23	0.04	0.01	
Duration of infertility	0.06	0.04	0.006	0.003	
Main effects					
Fear of intimacy		0.30**	0.19*	0.30**	
Moderator					
Mental toughness			0.21**	0.21**	
Interactions					
FI × MT				0.17*	
*R* ^2^	0.12**	0.18**	0.23**	0.26*	
Adjusted *R*^2^	0.11**	0.18**	0.21**	0.25*	
*F* change	17.48	29.38	26.87	10.12	

*p < 0.05, **p < 0.01., ***p < 0.001. FI, fear of intimacy; MT, mental toughness.

**Figure 2:**
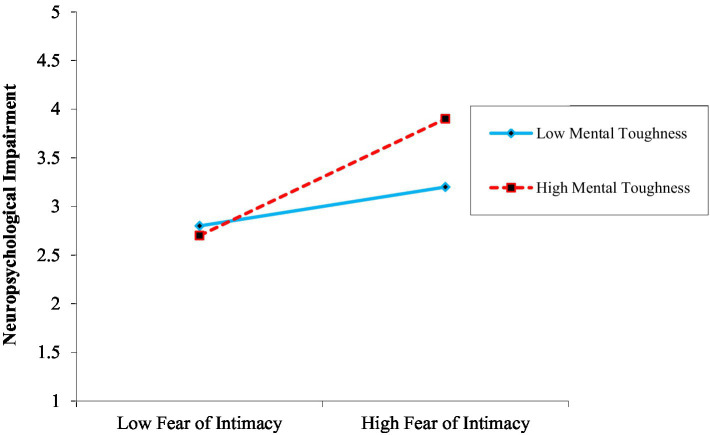
Moderating role of mental toughness on the link between fear of intimacy and neuropsychology impairment.

To examine of the moderating role of mental toughness on the association between fear of intimacy and neuropsychological impairment was presented in Table 4. We used sequentially test four models. Three control variables were entered into model 1; the findings indicated that the control variables are non-significant. The outcomes of model 2, took control variables and intimacy fear into account. With an R2 of 0.18 (*F* = 26.73, *p* < 0.01), the outcomes demonstrated that the explained variation was significant. Model 3 has mental toughness as a moderator. The outcomes demonstrated that the explained variance was significant with an R2 of 0.23 (*F* = 15.98, *p* < 0.01). Model 4 has an interaction term. With an R2 of 0.26* (*F* = 12.42, *p* < 0.05), the results indicated that explained variance is also statistically significant. Overall, the association between fear of intimacy and neuropsychological impairment is significantly moderated by mental toughness. Similar to this, the interaction plot (see Figure 2) using mean centering demonstrated that this association was weaker at the greater level of mental toughness (*β* = −0.04, *p* = ns) nonsignificant as opposed to the lower level (*β* = 0.43, *p* < 0.001). According to this, patients with a high level of mental toughness experience less intimacy-related fear and sense less neuropsychological impairment. The interaction’s pattern is consistent with H5.

## Discussion

Unwanted childlessness may have detrimental effects on the psychological health of infertile people. Infertile patients’ high anxiety, low self-esteem, mood swings, or sadness have all been linked to unfulfilled child wishes (68–73). Even though other scholars have looked into the influence of infertility on individuals’ psychological wellbeing, resilience, family or social support, and mental problem such as depression, anxiety, stress, etc. in the context of gender difference (74–76), whereas this study differed in that it studied the influence of fertility on neuropsychological prospects as well as intimacy-related problem in the context of male infertile patients in a developing country (i.e., Pakistan) by looking at unmentioned variables such as age, education, family structure, mode of treatment, duration of infertility and professional perspectives of just male patients because male infertility has gone almost ignored and unmentioned in the psychoanalytic literature (77).

Past studies were struck by the disparity between the extensive psychological research on infertility in women and the remarkable lack of material on men’s emotional and neuropsychological experiences with infertility (78). Even though reproductive endocrinologists agree that male factor infertility accounts for around 45% of all cases of infertility, this disparity persists (79). Similarly, after looking into the research history, no research work on male infertility has been done in Pakistan while in our patriarchic society male plays a significant role in society and male infertility is a large portion of our population. Similarly, unfortunately in our society women are blamed and the illusion that women are the root cause of infertility without any scientific evidence. The outcomes of this study are largely determined by two aspects one is the cognitive aspect of infertility (neuropsychological and mental toughness) and the second one that women are not only a root cause of infertility.

This article investigated the direct and indirect linkages between fear of intimacy and neuropsychological impairment of infertile patients by interacting and mediating the effect of QoL as well as moderating the effect of mental toughness. The majority of hypotheses were found to be supported in this study’s findings. The finding revealed a significant relationship of fear of intimacy with neuropsychological impairment and a significantly negative relationship with QoL. Furthermore, neuropsychological impairment has a negative relationship with QoL. The overall result of H1 was unique from previous studies (80–82) in the related field. Furthermore, the result showed that QoL significantly mediates the relationship between fear of intimacy and neuropsychological impairment. This finding was challenged by the view of Vo et al. (83) who argued that QoL significantly depends on the cognitive illness. Furthermore, the current research found that mental toughness buffered the influence of fear of intimacy on neuropsychological impairment. This means that fear of intimacy can be reduced by increasing mental toughness, thereby reducing neuropsychological impairment. Results revealed that infertile patients with mental toughness level are likely to neuropsychological impairment because they try to meet cognitive stress higher than optimal level. It is believed that that infertility status reveals a loss of mental toughness (mental control, commitment, and confidences).

Furthermore, in keeping with previous research, infertile men who have nuclear family structure scored significantly higher than infertile patients who have jointed family structure on fear of intimacy, poof QoL and neuropsychological impairment scores as seen in Table 5 which were indirectly associated with Aasen Nilsen et al. (84). Furthermore, results revealed that the infertile patients who were getting treatment from government hospitals had significantly high levels of fear of intimacy, poor mental tough, neuropsychological impairment, and poor QoL in comparison to those who were getting private treatment method (see Table 5) which is also linked with previous researches (85, 86).

**Table 5:** Mean, standard deviation, *t*-test and one-way ANOVA of variance in fear of intimacy, mental toughness, QoL and NPI in the term of across the demographic variables.

	Nuclear		Joints				
Variables	*M*	SD	*M*	SD	*t* (3,12)	*P*	Cohen’s *d*
Fear of intimacy	63.37	9.43	67.21	7.45	−2.34*	0.02	0.45
Mental toughness	17.97	4.18	20.26	3.42	−3.11**	0.002	0.60
QoL	24.02	6.63	23.45	5.63	0.484	0.62	0.12
NPI	98.87	13.38	93.36	13.47	2.18*	0.03	0.41

	Govt. treatment		Private treatment				
Variables	*M*	SD	*M*	SD	*t* (3,12)	*p*	Cohen’s *d*
Fear of intimacy	66.84	6.70	63.04	10.21	2.38**	0.01	0.44
Mental toughness	18.07	4.15	19.55	3.85	−2.03*	0.04	0.36
QoL	25.03	6.40	22.69	5.95	2.07*	0.04	0.37
NPI	99.50	12.19	94.28	14.46	2.12*	0.03	0.40

	Adulthood		Middle adult		Older adult				
Variables	*M*	SD	*M*	SD	*M*	SD	*F* (2,11)	η ^2^	*Post-hoc*
Fear of intimacy	63.73	9.34	62.84	8.62	69.75	6.56	6.13	0.094	3 > 2 > 1
Mental toughness	19.67	4.17	17.10	3.74	19.71	3.52	5.80	0.090	2 > 1 > 3
QoL	25.09	7.57	22.28	4.57	21.96	4.31	5.78	0.089	1 > 2 > 3
NPI	93.24	15.99	98.53	11.12	100.96	10.24	3.57	0.057	3 > 2 > 1

QoL, quality of life; NPI, neuropsychological impairment. * *p* < 0.05, ** *p* < 0.01.

Similarly, other scholars had also verified that older adult patients had a higher mental illness (87–89). Similarly, the researcher investigated older adults who have impoverished QoL because they suffer from fear of loneliness and hopelessness as well as cognitive problems (90). This finding is consistent with other studies that were carried out in Ghana, Iraq, and Germany, which also indicated that older adults scored high in intimacy-related problems (91, 92) and cognitive illness (93, 94) as well as poor QoL (95).

However, there are significant gaps in our knowledge of the impact of fear of intimacy on neuropsychological impairment in infertile patients, as well as the consequences of its negative effects on mental toughness and quality of life. In this context, the current study produced novel findings that investigate the impact of infertility patients’ fear of intimacy on neuropsychological impairment as well as QoL. The findings of this study also add to our understanding of the negative link between neuropsychological impairment and QoL. Furthermore, this study has observed sensitive details about how to reduce the negative effects of infernality on patients. Furthermore, the introduction of a moderator and mediator, as well as the use of a time lag approach (96, 97), allowed for a more comprehensive understanding of the impact of fear of intimacy on patient neuropsychological impairment, especially in the infertility dolman.

### Theoretical implications of the study

This study continues the tradition of infertility health behavior and clinical research into mental illness, fear of intimacy, poor mental toughness and QoL, and neuropsychological impairment. It investigates these notions in an understudied environment, namely, how fear of intimacy during infertile working hours might have negative consequences and influence neuropsychological functioning.

This publication, on the other hand, disseminates research by delving deeper into how fear of intimacy and mental toughness generate poor quality of life and how these negative outcomes may induce cognitive impairment. The findings of this study also contribute to the dissemination of previous and existing research by emphasizing the potential buffering of mental toughness on the link between fear of intimacy and neuropsychological impairment. Even though previous substantial clinical investigations on mental toughness neglected potential buffers, several recent studies have also avoided testing the moderators of mental toughness on outcome variables (psychological impairment).

The study’s findings suggest that MT can help reduce the impact of fear of intimacy on cognitive impairment. This study also disseminates the findings of recent studies that suggest that closeness about difficulties itself has a negative impact on QoL in the setting of infertile patients. This study used a multi-level method for purposive as well as experience sampling investigations, which is uncommon in the mental health literature.

### Limitations of the study

There are certain limitations to this study that should be considered. First, the cross-sectional design makes it difficult to draw a causal conclusion between research variables.

Second, our study only included infertile patients from one province’s infertility clinics. As a result, the sample may have an impact on the generalization of the results.

In summary, the current study is an attempt to understand how fear of intimacy, as an important aspect of personality, makes infertile patients more vulnerable to neuropsychological impairments, poor QoL, and poor mental toughness. The primary advantage of investigating the relationship between fear of intimacy and neuropsychological impairment is that it informs providers about the additional assessment needs of infertile patients.

In addition, the researchers propose that qualitative research utilizing narrative analysis or an interpretive phenomenological method may be used to gather real-world data to support the positivist approach used in this study. The research was only conducted in a relational and cross-sectional environment due to the pandemic’s unfavorable effects. Data were obtained physically, using the same logic and a more basic sampling technique. These should be taken into account while interpreting study results.

In this context, research involving infertile men patients who have been infected with the infertility and have recovered is believed to be necessary. Furthermore, adopting multimethod or mixed methods research in terms of data diversity is thought to produce substantial results in terms of external validity. Furthermore, research focused on cross-national comparisons is believed to yield crucial findings in terms of comprehending the nature of the problem.

## Conclusion

This study concluded that amongst infertile patients, fear of intimacy was significantly associated with neuropsychological impairment as well as fear of intimacy has positive significant association with emotional problems, learning problems, sensory and motor problem, concentration problem, mental & physical in coordination and depression. Similarly, fear of intimacy was negatively associated with quality of life. Furthermore, QoL was significantly mediated by the association between fear of intimacy and neuropsychological impairment. Moreover, mental toughness moderated the association between fear of intimacy and neuropsychological impairment.

## Data availability statement

The raw data supporting the conclusions of this article will be made available by the authors, without undue reservation.

## Ethics statement

Study was approved by the Islamic International University ethics committee and consent was obtained from the participants. Moreover study has adapted all the guidelines outlined in the declaration of Helsinki ethical principles.

## Author contributions

SH, MB, and NK executed the project and wrote the manuscript. SH, SF, and SB had literature reviewed, and data collection. EJ contributed to project administration, funding, and statistical analysis. All authors contributed to the article and approved the submitted version.

## Funding

The authors acknowledged the financial support from the Postgraduate Education Reform and Quality Improvement Project of Henan Province (No.YJS2022KC30); the Postgraduate Cultivating Innovation and Quality Improvement Action Plan of Henan University (No.YJSJG2022XJ059).

## Conflict of interest

The authors declare that the research was conducted in the absence of any commercial or financial relationships that could be construed as a potential conflict of interest.

## Publisher’s note

All claims expressed in this article are solely those of the authors and do not necessarily represent those of their affiliated organizations, or those of the publisher, the editors and the reviewers. Any product that may be evaluated in this article, or claim that may be made by its manufacturer, is not guaranteed or endorsed by the publisher.

## References

[1] Kopa Z Keszthelyi M Sofikitis N. Administration of antioxidants in the infertile male: when it may have a beneficial effect? Curr Pharm Des. (2021) 27:2665–8. doi: 10.2174/1381612826666200303115552, PMID: 32124691

[2] Zegers-Hochschild F Adamson GD Dyer S Racowsky C De Mouzon J Sokol R. The international glossary on infertility and fertility care, 2017. Hum Reprod. (2017) 32:1786–801. doi: 10.1093/humrep/dex234, PMID: 29117321 PMC5850297

[3] Vander Borght M Wyns C. Fertility and infertility: definition and epidemiology. Clin Biochem. (2018) 62:2–10. doi: 10.1016/j.clinbiochem.2018.03.012 29555319

[4] Thoma ME McLain AC Louis JF King RB Trumble AC Sundaram R. Prevalence of infertility in the United States as estimated by the current duration approach and a traditional constructed approach. Fertil Steril. (2013) 99:1324–1331.e1. doi: 10.1016/j.fertnstert.2012.11.037, PMID: 23290741 PMC3615032

[5] Slama R Hansen OKH Ducot B Bohet A Sorensen D Giorgis Allemand L. Estimation of the frequency of involuntary infertility on a nation-wide basis. Hum Reprod. (2012) 27:1489–98. doi: 10.1093/humrep/des070, PMID: 22416008

[6] Zhou Z Zheng D Wu H Li R Xu S Kang Y. Epidemiology of infertility in China: a population-based study. BJOG. (2018) 125:432–41. doi: 10.1111/1471-0528.14966 29030908

[7] Inhorn MC Patrizio P. Infertility around the globe: new thinking on gender, reproductive technologies and global movements in the 21st century. Hum Reprod Update. (2015) 21:411–26. doi: 10.1093/humupd/dmv016, PMID: 25801630

[8] Poornima S Daram S Devaki RK Qurratulain H. Chromosomal abnormalities in couples with primary and secondary infertility: genetic counseling for assisted reproductive techniques (ART). J Reprod Infertil. (2020) 4:269. doi: 10.18502/jri.v21i4.4331 PMC7648875 33209743

[9] Kumar N Singh AK. Trends of male factor infertility, an important cause of infertility: a review of literature. J Hum Reprod Sci. (2015) 8:191–6. doi: 10.4103/0974-1208.170370, PMID: 26752853 PMC4691969

[10] Bai CF Sun JW Li J Jing WH Zhang XK Zhang X. Gender differences in factors associated with depression in infertility patients. J Adv Nurs. (2019) 75:3515–24. doi: 10.1111/jan.14171, PMID: 31410867

[11] Ussher JM Perz J. Threat of biographical disruption: the gendered construction and experience of infertility following cancer for women and men. BMC Cancer. (2018) 18:1–17. doi: 10.1186/s12885-018-4172-5 29506492 PMC5836444

[12] Alhathal N Maddirevula S Coskun S Alali H Assoum M Morris T. A genomics approach to male infertility. Genet Med. (2020) 22:1967–75. doi: 10.1038/s41436-020-0916-0 32719396

[13] Wang C Horby PW Hayden FG Gao GF. A novel coronavirus outbreak of global health concern. Lancet. (2020) 395:470–3. doi: 10.1016/S0140-6736(20)30185-9, PMID: 31986257 PMC7135038

[14] Liu C Li W Chen X Liu M Zuo L Chen L. Dose-response association between transportation noise exposure and type 2 diabetes: a systematic review and meta-analysis of prospective cohort studies. Diabetes Metab Res Rev. (2023) 39:e3595. doi: 10.1002/dmrr.3595, PMID: 36408740

[15] Liu M Kamper-DeMarco KE Zhang J Xiao J Dong D Xue P. Time spent on social media and risk of depression in adolescents: a dose–response meta-analysis. Int J Environ Res Public Health. (2022) 19:5164. doi: 10.3390/ijerph19095164, PMID: 35564559 PMC9103874

[16] Volgsten H Skoog Svanberg A Ekselius L Lundkvist Ö Sundström Poromaa I. Prevalence of psychiatric disorders in infertile women and men undergoing in vitro fertilization treatment. Hum Reprod. (2008) 23:2056–63. doi: 10.1093/humrep/den154, PMID: 18583334 PMC2517152

[17] Volgsten H Svanberg AS Ekselius L Lundkvist Ö Poromaa IS. Risk factors for psychiatric disorders in infertile women and men undergoing in vitro fertilization treatment. Fertil Steril. (2010) 93:1088–96. doi: 10.1016/j.fertnstert.2008.11.008, PMID: 19118826

[18] Biggs A. Brough P. Drummond S.. Lazarus and Folkman’s psychological stress and coping theory in the handbook of stress and health: a guide to research and practice, eds. C. L. Cooper and J. C. Quick (2017) Wiley Blackwell. doi: 10.1002/9781118993811.ch21

[19] Lazarus RS. Toward better research on stress and coping. Am Psychol. (2000) 55:665–73. doi: 10.1037/0003-066X.55.6.665 10892209

[20] Lazarus RS Folkman S. Stress, appraisal, and coping. New York, NY. Springer Publishing Company (1984).

[21] Aghakhani N Ewalds-Kvist BM Sheikhan F Khoei EM. Iranian women’s experiences of infertility: a qualitative study. Int J Reprod Biomed. (2020) 18:65–73. doi: 10.18502/ijrm.v18i1.6203 32043073 PMC6996123

[22] Bhamani SS Zahid N Zahid W Farooq S Sachwani S Chapman M. Association of depression and resilience with fertility quality of life among patients presenting to the infertility centre for treatment in Karachi Pakistan. BMC Public Health. (2020) 20:1–11. doi: 10.1186/s12889-020-09706-1 33097027 PMC7585180

[23] Onofre J Geenen L Cox A Van Der Auwera I Willendrup F Andersen E. Simplified sperm testing devices: a possible tool to overcome lack of accessibility and inconsistency in male factor infertility diagnosis. An opportunity for low-and middle-income countries. Facts Views Vis Obgyn. (2021) 13:79–93. doi: 10.52054/FVVO.13.1.011, PMID: 33889864 PMC8051200

[24] McCleary-Sills J Namy S Nyoni J Rweyemamu D Salvatory A Teven E. Stigma, shame and women’s limited agency in help-seeking for intimate partner violence. Glob Public Health. (2016) 11:224–35. doi: 10.1080/17441692.2015.1047391, PMID: 26156577

[25] Roberts L Renati S Solomon S Montgomery S. Women and infertility in a pronatalist culture: mental health in the slums of Mumbai. Int J Women’s Health. (2020) 12:993–1003. doi: 10.2147/IJWH.S273149, PMID: 33192102 PMC7654515

[26] Mansour F Mohdy HA. Intimate partner violence among women with female infertility. Am J Nurs. (2018) 6:309–16.

[27] Çambel B Akköz Çevik S. Prevalence of intimate partner and family violence among women attending infertility clinic and relationship between violence and quality of life. J Obstet Gynaecol. (2022) 42:2082–8. doi: 10.1080/01443615.2021.2024156 35068321

[28] Willer EK. Running-in (to) transition: embodied practice under the load of infertility, baby loss, and motherhood. Health Commun. (2021) 36:1176–87. doi: 10.1080/10410236.2020.1748830, PMID: 32312083

[29] Grunberg PH Da Costa D Dennis CL O’Connell S Lahuec A Zelkowitz P. How did you cope with such concerns?’: insights from a monitored online infertility peer support forum. Hum Fertil. (2021):1–15. doi: 10.1080/14647273.2021.1959952, PMID: 34347545

[30] Gerlock AA Grimesey J Sayre G. Military-related posttraumatic stress disorder and intimate relationship behaviors: a developing dyadic relationship model. J Marital Fam Ther. (2014) 40:344–56. doi: 10.1111/jmft.12017, PMID: 24749950

[31] Li Y Zhang X Shi M Guo S Wang L. Resilience acts as a moderator in the relationship between infertility-related stress and fertility quality of life among women with infertility: a cross-sectional study. Health Qual Life Outcomes. (2019) 17:1–9. 30770738 10.1186/s12955-019-1099-8 PMC6377764

[32] Cha DS Carmona NE Subramaniapillai M Mansur RB Lee Y Lee JH. Cognitive impairment as measured by the THINC-integrated tool (THINC-it): association with psychosocial function in major depressive disorder. J Affect Disord. (2017) 222:14–20. doi: 10.1016/j.jad.2017.06.036, PMID: 28667888

[33] Jahromi BN Mansouri M Forouhari S Poordast T Salehi A. Quality of life and its influencing factors of couples referred to an infertility center in Shiraz, Iran. Int J Fertil Steril. (2018) 11:293. doi: 10.22074/ijfs.2018.5123 29043705 PMC5641461

[34] Lakatos E Szigeti JF Ujma PP Sexty R Balog P. Anxiety and depression among infertile women: a cross-sectional survey from Hungary. BMC Womens Health. (2017) 17:1–9. doi: 10.1186/s12905-017-0410-2 28738833 PMC5525318

[35] Evers K Maljaars J Schepens H Vanaken GJ Noens I. Conceptualization of quality of life in autistic individuals. Dev Med Child Neurol. (2022) 64:950–6. doi: 10.1111/dmcn.15205, PMID: 35323990

[36] Masoumi SZ Garousian M Khani S Oliaei SR Shayan A. Comparison of quality of life, sexual satisfaction and marital satisfaction between fertile and infertile couples. Int J Fertil Steril. (2016) 10:290–6. PMID: 27695611 10.22074/ijfs.2016.5045 PMC5023039

[37] Namdar A Naghizadeh MM Zamani M Yaghmaei F Sameni MH. Quality of life and general health of infertile women. Health Qual Life Outcomes. (2017) 15:139. doi: 10.1186/s12955-017-0712-y, PMID: 28701163 PMC5508693

[38] Gameiro S Boivin J Peronace L Verhaak CM. Why do patients discontinue fertility treatment? A systematic review of reasons and predictors of discontinuation in fertility treatment. Hum Reprod Update. (2012) 18:652–69. doi: 10.1093/humupd/dms031, PMID: 22869759 PMC3461967

[39] Li G Zhao D Wang Q Zhou M Kong L Fang M. Infertility-related stress and quality of life among infertile women with polycystic ovary syndrome: does body mass index matter? J Psychosom Res. (2022) 158:110908. doi: 10.1016/j.jpsychores.2022.110908 35421758

[40] Patterson P Perz J Tindle R McDonald FE Ussher JM. Infertility after cancer: how the need to be a parent, fertility-related social concern, and acceptance of illness influence quality of life. Cancer Nurs. (2021) 44:E244–51. doi: 10.1097/NCC.0000000000000811 32209862

[41] Zurlo MC Della Volta MFC Vallone F. Predictors of quality of life and psychological health in infertile couples: the moderating role of duration of infertility. Qual Life Res. (2018) 27:945–54. doi: 10.1007/s11136-017-1781-4, PMID: 29307056

[42] Budarin YS Chirkov DK Magomedov FB. Digital cooperation in the Russian Federation In: Bogoviz, A.V., Suglobov, A.E., Maloletko, A.N., Kaurova, O.V. (eds). Сooperation and sustainable development. Cham: Springer (2022). 27–38.

[43] St Clair-Thompson H Bugler M Robinson J Clough P McGeown SP Perry J. Mental toughness in education: exploring relationships with attainment, attendance, behaviour and peer relationships. Educ Psychol. (2015) 35:886–907. doi: 10.1080/01443410.2014.895294

[44] Brand S Kalak N Gerber M Clough PJ Lemola S Pühse U. During early and mid-adolescence, greater mental toughness is related to increased sleep quality and quality of life. J Health Psychol. (2016) 21:905–15. doi: 10.1177/1359105314542816, PMID: 25060987

[45] Valle MF Huebner ES Suldo SM. An analysis of hope as a psychological strength. J Sch Psychol. (2006) 44:393–406. doi: 10.1016/j.jsp.2006.03.005

[46] Clough P Strycharczyk D. Developing mental toughness: improving performance, wellbeing and positive behaviour in others. London: Kogan Page Publishers (2012).

[47] Gerber M Feldmeth AK Lang C Brand S Elliot C Holsboer-Trachsler E. The relationship between mental toughness, stress, and burnout among adolescents: a longitudinal study with Swiss vocational students. Psychol Rep. (2015) 117:703–23. doi: 10.2466/14.02.PR0.117c29z6, PMID: 26652888

[48] Lin Y Mutz J Clough PJ Papageorgiou KA. Mental toughness and individual differences in learning, educational and work performance, psychological well-being, and personality: a systematic review. Front Psychol. (2017) 8:1345. doi: 10.3389/fpsyg.2017.01345, PMID: 28848466 PMC5554528

[49] Jones MI Parker JK. Mindfulness mediates the relationship between mental toughness and pain catastrophizing in cyclists. Eur J Sport Sci. (2018) 18:872–81. doi: 10.1080/17461391.2018.1478450, PMID: 29870312

[50] Mojtahedi D Dagnall N Denovan A Clough P Hull S Canning D. The relationship between mental toughness, job loss, and mental health issues during the COVID-19 pandemic. Front Psych. (2021) 11:607246. doi: 10.3389/fpsyt.2020.607246, PMID: 33613333 PMC7886783

[51] Lau Y Chan KS. Psychometric evaluation of the Chinese version of the fear of intimacy with helping professionals scale. PLoS One. (2018) 13:e0196774. doi: 10.1371/journal.pone.0196774, PMID: 29795563 PMC5967800

[52] Papageorgiou KA Malanchini M Denovan A Clough PJ Shakeshaft N Schofield K. Longitudinal associations between narcissism, mental toughness and school achievement. Personal Individ Differ. (2018) 131:105–10. doi: 10.1016/j.paid.2018.04.024

[53] Khan MNS Akhter MS Ayub M Alam S Laghari N. Translation and validation of WHOQOL BREF. J Coll of Physicians Surg Pak. (2003) 13:98–100. 12685953

[54] World Health Organization. WHOQOL-BREF: introduction, administration, scoring and generic version of the assessment: field trial version, December (No. WHOQOL-BREF) (1996).

[55] Naheed SR. Neuropsychopathology and role of family support in improvement of the stroke patient. Unpublished Ph.D Disertation, National Institute of Psychology, QAU, Islamabad (2000).

[56] Anderson JC Gerbing DW. Structural equation modeling in practice: a review and recommended two-step approach. Psychol Bull. (1988) 103:411–23. doi: 10.1037/0033-2909.103.3.411

[57] Podsakoff PM MacKenzie SB Lee JY Podsakoff NP. Common method biases in behavioral research: a critical review of the literature and recommended remedies. J Appl Psychol. (2003) 88:879–903. doi: 10.1037/0021-9010.88.5.879, PMID: 14516251

[58] Chen Y Hou S Fu K Han X Ye L. Low-velocity impact response of composite sandwich structures: modelling and experiment. Compos Struct. (2017) 168:322–34. doi: 10.1016/j.compstruct.2017.02.064

[59] Sellbom M Tellegen A. Factor analysis in psychological assessment research: common pitfalls and recommendations. Psychol Assess. (2019) 31:1428–41. doi: 10.1037/pas0000623, PMID: 31120298

[60] Khan NA Khan AN Moin MF Pitafi AH. A trail of chaos: how psychopathic leadership influence employee satisfaction and turnover intention via self-efficacy in tourism enterprises. J Leis Res. (2021) 52:3:347–369. doi: 10.1080/00222216.2020.1785359

[61] Fornell C Larcker DF. Structural equation models with unobservable variables and measurement error: algebra and statistics. J Mark Res. (1981) 18:382. doi: 10.2307/3150980

[62] Cao H Zhang H Wang C Zhang B. Bernstein. (2019) 11:786. doi: 10.3390/w11040786

[63] Nunnally JC Bernstein IH. Psychological theory. (New York, NY: MacGraw-Hill). (1994).

[64] Bagozzi RP. Further thoughts on the validity of measures of elation, gladness, and joy. J Pers Soc Psychol. (1991) 61:98–104. doi: 10.1037/0022-3514.61.1.98

[65] Khan NH Hassan S Bahader S Fatima S Zaidi SMIH Virk R. How daily obstacles affect frontline healthcare professionals’ mental health during omicron: a daily diary study of handwashing behavior. Int J Environ Res Public Health. (2022) 19:8748. doi: 10.3390/ijerph19148748, PMID: 35886597 PMC9320559

[66] Tripp CP Hair ML. An infrared study of the reaction of octadecyltrichlorosilane with silica. Langmuir. (1992) 8:1120–6. doi: 10.1021/la00040a018

[67] Milman E Neimeyer RA Fitzpatrick M MacKinnon CJ Muis KR Cohen SR. Prolonged grief and the disruption of meaning: establishing a mediation model. J Couns Psychol. (2019) 66:714–25. doi: 10.1037/cou0000370, PMID: 31647284

[68] Fisher JR Hammarberg K. Psychological and social aspects of infertility in men: an overview of the evidence and implications for psychologically informed clinical care and future research. Asian J Androl. (2012) 14:121–9. doi: 10.1038/aja.2011.72, PMID: 22179515 PMC3735147

[69] Holley SR Pasch LA Bleil ME Gregorich S Katz PK Adler NE. Prevalence and predictors of major depressive disorder for fertility treatment patients and their partners. Fertil Steril. (2015) 103:1332–9. doi: 10.1016/j.fertnstert.2015.02.018, PMID: 25796319 PMC4417384

[70] Li X Zhang X Jia T. Humanization of nature: testing the influences of urban park characteristics and psychological factors on collegers’ perceived restoration. Urban For Urban Green. (2023) 79:127806. doi: 10.1016/j.ufug.2022.127806

[71] Lyu Z Feng X Li N Zhao W Wei L Chen Y. Human papillomavirus in semen and the risk for male infertility: a systematic review and meta-analysis. BMC Infect Dis. (2017) 17:1–9. doi: 10.1186/s12879-017-2812-z 29121862 PMC5679371

[72] Schick M Rösner S Toth B Strowitzki T Wischmann T. Exploring involuntary childlessness in men–a qualitative study assessing quality of life, role aspects and control beliefs in men’s perception of the fertility treatment process. Hum Fertil. (2016) 19:32–42. doi: 10.3109/14647273.2016.1154193, PMID: 27007070

[73] Wischmann T Thorn P. (male) infertility: what does it mean to men? New evidence from quantitative and qualitative studies. Reprod Biomed Online. (2013) 27:236–43. doi: 10.1016/j.rbmo.2013.06.002, PMID: 23876974

[74] Cao W Fang Z Hou G Han M Xu X Dong J. The psychological impact of the COVID-19 epidemic on college students in China. Psychiatry Res. (2020) 287:112934. doi: 10.1016/j.psychres.2020.112934, PMID: 32229390 PMC7102633

[75] Kumar P Kumar N Aggarwal P Yeap JA. Working in lockdown: the relationship between COVID-19 induced work stressors, job performance, distress, and life satisfaction. Curr Psychol. (2021) 40:6308–6323. doi: 10.1007/s12144-021-01567-0 33746462 PMC7955899

[76] Zheng F Khan NA Hussain S. The COVID 19 pandemic and digital higher education: exploring the impact of proactive personality on social capital through internet self-efficacy and online interaction quality. Child Youth Serv Rev. (2020) 119:105694–12. doi: 10.1016/j.childyouth.2020.105694

[77] Keylor R Apfel R. Male infertility: integrating an old psychoanalytic story with the research literature. Stud Gend Sex. (2010) 11:60–77. doi: 10.1080/15240651003666326

[78] Nagórska M Bartosiewicz A Obrzut B Darmochwał-Kolarz D. Gender differences in the experience of infertility concerning polish couples: preliminary research. Int J Environ Res Public Health. (2019) 16:23–37. doi: 10.3390/ijerph16132337 PMC6651646 31269703

[79] Babul-Hirji R Hirji R Chitayat D. Genetic counselling for infertile men of known and unknown etiology. Transl Androl Urol. (2021) 10:1479–85. doi: 10.21037/tau-2019-gcmi-09, PMID: 33850782 PMC8039627

[80] Halcomb L. Men and infertility: insights from the sociology of gender. Sociol Compass. (2018) 12:e12624. doi: 10.1111/soc4.12624

[81] Hanna E Gough B. The impact of infertility on men’s work and finances: findings from a qualitative questionnaire study. Gend Work Organ. (2020) 27:581–91. doi: 10.1111/gwao.12414

[82] Jamil S Shoaib M Aziz W Ather MH. Does male factor infertility impact on self-esteem and sexual relationship? Andrologia. (2020) 52:e13460. doi: 10.1111/and.13460, PMID: 31691340

[83] Vo THM Nakamura K Seino K Nguyen HTL Van Vo T. Fear of falling and cognitive impairment in elderly with different social support levels: findings from a community survey in Central Vietnam. BMC Geriatr. (2020) 20:1–10. doi: 10.1186/s12877-020-01533-8 PMC7164140 32299392

[84] Aasen Nilsen S Breivik K Wold B Bøe T. Divorce and family structure in Norway: associations with adolescent mental health. J Divorce Remarriage. (2018) 59:175–94. doi: 10.1080/10502556.2017.1402655

[85] Corrigan PW Nieweglowski K. How does familiarity impact the stigma of mental illness? Clin Psychol Rev. (2019) 70:40–50. doi: 10.1016/j.cpr.2019.02.001 30908990

[86] St. Vil NM St. Vil C Fairfax CN. Posttraumatic slave syndrome, the patriarchal nuclear family structure, and African American male–female relationships. Soc Work. (2019) 64:139–46. doi: 10.1093/sw/swz002, PMID: 30722067

[87] Conejero I Olié E Courtet P Calati R. Suicide in older adults: current perspectives. Clin Interv Aging. (2018) 13:691–9. doi: 10.2147/CIA.S130670, PMID: 29719381 PMC5916258

[88] Mukhtar S. Psychological impact of COVID-19 on older adults. Curr Med Res Pract. (2020) 10:201–2. doi: 10.1016/j.cmrp.2020.07.016, PMID: 32839732 PMC7373678

[89] Taylor HO Taylor RJ Nguyen AW Chatters L. Social isolation, depression, and psychological distress among older adults. J Aging Health. (2018) 30:229–46. doi: 10.1177/0898264316673511, PMID: 28553785 PMC5449253

[90] Ghoncheh KA Liu CH Lin CY Saffari M Griffiths MD Pakpour AH. Fear of COVID-19 and religious coping mediate the associations between religiosity and distress among older adults. Health Promot Perspect. (2021) 11:316–22. doi: 10.34172/hpp.2021.40, PMID: 34660226 PMC8501474

[91] Ganong K Larson E. Intimacy and belonging: the association between sexual activity and depression among older adults. Soc Ment Health. (2011) 1:153–72. doi: 10.1177/2156869311431612

[92] Rheaume C Mitty E. Sexuality and intimacy in older adults. Geriatr Nurs. (2008) 29:342–9. doi: 10.1016/j.gerinurse.2008.08.004 18929184

[93] Morris RG Worsley C Matthews D. Neuropsychological assessment in older people: old principles and new directions. Adv Psychiatr Treat. (2000) 6:362–70. doi: 10.1192/apt.6.5.362

[94] Rog LA Park LQ Harvey DJ Huang CJ Mackin S Farias ST. The independent contributions of cognitive impairment and neuropsychiatric symptoms to everyday function in older adults. Clin Neuropsychol. (2014) 28:215–36. doi: 10.1080/13854046.2013.876101, PMID: 24502686 PMC4021718

[95] Huang R Ghose B Tang S. Effect of financial stress on self-rereported health and quality of life among older adults in five developing countries: a cross sectional analysis of WHO-SAGE survey. BMC Geriatr. (2020) 20:1–12. doi: 10.1186/s12877-020-01687-5 PMC7425413 32787806

[96] Detert JR Burris ER. Leadership behavior and employee voice: is the door really open? Acad Manag J. (2007) 50:869–84. doi: 10.5465/amj.2007.26279183

[97] Pan Z-Y Zhong H-J Huang D-N Wu L-H He X-X. Beneficial effects of repeated washed microbiota transplantation in children with autism. Front Pediatr. (2022) 10:971. doi: 10.3389/fped.2022.928785 PMC9249087 35783298

